# Hypodermic Medication

**Published:** 1871-05

**Authors:** W. A. Greene

**Affiliations:** Americus, Ga.


### HYPODERMIC MEDICATION.

BY W. A. GREENE, M. D., OF AMERICUS, GA.

A few years since, I wrote an article on "Hypodermic administration of medicinal agents," which was published in the Atlanta Medical Journal, (May, 1867,) and so far as I know, was the first article on this subject published in any medical periodical of this country, and strange to say, I have not since seen the subject *practically* treated by any author.

Many of my professional friends then thought I extolled this method of administering medicines extravagantly. I have continued, however, to use the hypodermic syringe more extensively, and not only have no reason to change my views therein expressed, but am well satisfied I failed to do the subject justice.

It is a matter of surprise that some of the members of the medical profession entertain a prejudice against the employment of sub-cutaneous medication.

To account for it, I have sometimes thought it results from a lack of a little experience in the use of the hypodermic syringe. I am sure that when judiciously used, it is just as safe as medication *per anum*, *per os*, or *per* any other way, and is certainly much more rapid, constant and efficient in its effects. It not only relieves pain CERTAINLY, but it sometimes cures, and cures permanently and radically—yea, *frequently* does so. I am also surprised that even leading medical journals contain so little on this subject. *Ranking's Abstract* and the *American Journal of the Medical Sciences*, for the past three or four years, barely allude to it. The same may be said of the *Practitioner*, *Louisville and Richmond Journal*, *New Orleans Journal*, and all others I have had an opportunity of reading.

You have done me the honor to republish the above-mentioned article in your April number of the present year, for which I sincerely thank you. It is gratifying to know that my endeavors in this direction have been kindly received by the profession, and their appreciation manifested by requesting my further views upon the subject, based upon more extensive experience, and guided by the experience and discoveries of others, whose results I have watched and read with great interest.

It is my present purpose again to call attention to this important and interesting subject. I shall endeavor to be practical in what I write, giving no facts that are not sustained by experience, observation, or well authenticated, for I am well satisfied that much, perhaps most, that is published in our medical journals of the present day, contains too much speculative theory, and that too many medical men write for mere notoriety. Medical journals should be addressed to the requirements of physicians whose engagements do not permit them to search over the immense fields from which the fruit has been gathered; to practitioners in the country, as conveying the results of the active labors of the "toilers" in the profession. They deserve the attention and care of medical journals, at least to some extent, and I hope, Mess. Editors, you will not forget this in the management of your journal, now in its embryo state. Of course, I do not assume to lecture editors of medical journals, but I long to see them contain matter more practical, more instructive to the mass of doctors, and thereby more useful and valuable to the country.

The following points in Hypodermy are well established, and I invite particular attention to their consideration :

1st. Clear, neutral solutions alone should be used, as they rarely produce irritation.

2d. Whether administered hypodermically or by the mouth, or rectum. the chief physiological and therapeutical effects are the same *in kind*, yet varying *in degree.*

3d. Some drugs, when administered hypodermically or subcutaneously, produce certain symptoms which are absent when administered by other methods, and certain unpleasant symptoms are thus avoided, which would interdict the use of perhaps an indispensable treatment.

4th. Rapidity of absorption and intensity of effect, as a general rule, may always be anticipated.

5th. Locality of injection, whether near or remote from the point affected, as to effect or relief, makes no difference.

6th. Then the advantages to be derived from this mode of introducing drugs are (a) rapidity of action; (b) intensity of effect; (c) facility of introduction; (d) with some medicines, avoidance of unpleasant symptoms. Therefore, when rapid and decided effects of drugs are desired, this mode of administration ought to be adopted.

The medicines that have been principally employed by the Hypodermic method, are aconitine, the preparations of opium, atropia, quinine, strychnine, conia, podophyline, iodide of potassia, bichlorid of mercury, prussic acid, colocynth, aloes, veratrum viride, calabar bean, tr. cannabis indicæ, tr. hyosiamus, and ergotine. Those in general use, and whose effects are best understood are, quinine, morphine, atropine, veratrum, calabar bean, ergotine, and the bichlorid of mercury, the last experimentally, and its practical value not yet determined. I propose to speak of these practically, giving my observation of them in detail, and the diseases and conditions for which they have been prescribed.

*Quinine*—while I would not discard it from hypodermic use—must be prescribed with great caution. It often produces violent ulceration, destroying the tissue beneath the skin, and presenting to view the uncovered muscles, conveying to the mind the idea of the action of some corrosive poison. And it has produced this effect in my hands when simply suspended in water, without any of the dissolving acids. I attribute this unpleasant and unfortunate result of the use of quinine to its *great insolubility*—and hope some means may be discovered by which these unhappy effects may be overcome. Another very serious obstacle to its use, is the liability of producing tetanus, which has, however, never been the case in my own experience, but has occurred in the hands of others, of which I give a well-authenticated instance:

M. Arnold, a military surgeon at Constantia, (Algeria,) who had used quinine successfully in ague, warns the profession as to tetanus being induced by such injections, and mentions two cases—a child and an adult, who both died of traumatic tetanus from hypodermic injections of sulphate of quinine, dissolved in water and a little sulphuric acid. Both patients were suffering from ague. I would, therefore, recommend great caution in the indiscriminate use of this medicine hypodermically. There are, however, circumstances under which I would not hesitate to use it, and do use it. Where life is endangered by the recurrence of a congestive chill, and my patient will not tolerate quinine by the stomach or rectum, or can't swallow it or refuses to do so—or I have not *time* to wait for its constitutional effects by the ordinary method of administration, then, I repeat, I would not hesitate to take the *chances* of ulceration or tetanus. The following is the solution of quinine I employ:

| | | |
|---|---|---|
| ℞—Sulph. quinine, | . . | gr. xxx. |
| Sulph. acid *dilut.* | . . | gtt. x. |
| Water (boiling) | . . | ℥ ss. M. |

Sig.—Inject from one half to one drachm half hour before the paroxysm.

The preparations of *opium* and *belladonna* deserve special mention, because of their universal and almost unlimited use by the hypodermic method for the relief of pain and many of the distressing symptoms of disease, the effects of which are as satisfactory as they are astounding. While diseases differ from each other materially, in cause, symptoms and severity, there is one characteristic in common, which is *pain*, ever present and harassing—at the bedside by day, unappeased at night—a phantom that walks by the side of the physician, ever ready to bar the door of the sick chamber against his entrance. In these preparations, we possess therapeutic weapons that speedily overcome and vanquish this dread enemy.

In my former article, I recommended the use of the acetate of morphine, but now use and prefer the sulphate, because it is more soluble, and consequently, less irritating.

The following is my solution :

| | | |
|---|---|---|
| ℞—Sulph. morphia, | . . | gr. xviii. |
| Sulph. atropia, | . . | gr. i. |
| Carbolic acid, | . . | gr. $\frac{1}{3}$. |
| Water, | . . | ℥ i. M. |

Sig.—Ten drops equal to one half grain of morphine, and one thirty-sixth grain of atropia.

The carbolic acid produces a perfect solution without the further addition of other acids, and prevents the cryptogamic deposit or growth that you find in the ordinary solutions of morphine without it—thus enabling us to keep a larger quantity of the solution on hand, though it is best to use a fresh solution whenever practicable.

The number of drops calculated for an ounce is three hundred and sixty (360,) and not four hundred and eighty (480) as ordinarily supposed; the test of the drops being the amount that passes off as such from the point of the hypodermic needle. There ought to be a more reliable standard of the dross than now exists, and the needles of the syringe should always be the same size. Those manufactured by Lier and Charrier, of Paris, are of precisely the same size—also those prepared by Tieman & Co., of New York. I use the *sulphate* of atropia, because it is the most soluble, and equally as efficacious. As will be perceived, I combine the atropia in this solution for obvious reasons. The question of antagonism between opium and belladonna and their preparations, has excited considerable controversy during the past few years. I have not time to enter into a full discussion of this question, but will mention that it seems to be the result of extensive observation by learned men of the profession : that morphine and atropine are naturally opposing poisons; but that while, on the one hand, atropine diminishes or removes the hurtful influence of morphine on the brain, without diminishing its sedative and pain-allaying power, yet on the other there is no complete antagonism between the two agents over the whole range of their action. In certain functions of the organism, the antagonism is incomplete—in others it is wholly absent, and in respect to the urinary bladder, the effects of the two poisons are alike. It seems, then, that the evidence of antagonism is inconclusive, yet, in my opinion, the physician who does not administer the one in case of poisoning by the other, *fails to do his duty.* The success depends upon the prompt administration of a dose or amount adequate to the case in hand; and there can be no other rational guide to this than the quantity of poison taken and probably absorbed, as evidenced by existing symptoms. The combination of the atropine in the morphine solution seems to be valuable, then, in many respects. The essential effects of opium are both intensified and prolonged by the concurrent action of belladonna. It prevents the nausea frequently, that would otherwise follow the use of morphine. This combination, as a general diffusable stimulant, surpasses all other drugs, and is a most appropriate and hopeful therapeutic means in all diseases where there is failure of the heart's action, i. e. depression of the sympathetic nerve force. It must be borne in mind that an excessive dose will produce depression from over stimulation. Given with a view of exciting or sustaining the heart's action, the dose will range from the 1-100 to the 1-60 of a grain of atropia, and should never exceed the 1-40. It is especially in cases of the neuralgias—*of pains of every character*, that the administration of this sedative exhibits to most advantage. The effects of a few drops of this solution in a case of facial neuralgia or hepatic colic, for instance, injected beneath the skin of the forearm into the areolar tissue, are sometimes almost marvellous; usually within ten minutes, the patient is quite free from pain. The effect of one such dose is startling to any one who has not had some experience in these ca es—the beneficial influence continuing for several hours, and in ordinary cases, not arising from organic disease, effecting a cure. It is remarkable that morphine thus used, has a more *permanent* effect in allaying pain than when given in any other way. After a fracture has been reduced, and the limb placed in a proper position, a small dose injected into the areolar tissue of the limb is of great value in preventing muscular spasm, and, I think, ought never to be neglected. In the distressing vomiting from seasickness, which sometimes imperils life, I imagine this solution injected over the region of the stomach, would produce charming relief, as it certainly does in other vomitings. I have seen hiccough complicating pneumonia, which had resisted for days, various treatment, and strongly threatened death, instantly relieved by injecting six drops of this solution into the intercostal region. Puerperal convulsions after labor are almost always relieved by the injection of ten drops in the arm. In the distressing paroxysms of asthma, I have never found any remedy so efficacious. In obstetric practice, I find the sub-cutaneous injection of this solution very useful under the following circumstances and conditions, viz:

1st. During painful dilatation and expulsory periods, especially in primiparæ and in narrow pelvis. 2d. In spasmodic pains. 3d. In painful complications of the process of labor in general. 4th. In severe after-pains. It is in the first category of cases that I have especially employed it, injecting one or both thighs. I might go on and occupy pages, enumerating the conditions and circumstances where these drugs have been employed with complete satisfaction. I will only further state that wherever *pain* is present, which no human being ever willingly endured for a single moment, always use the hypodermic syringe. In children and delicate females I employ the compound liquor of opium prepared by Dr. Edward R. Squibbs, in doses from five to fifteen drops, or more, always beginning with a minimum, and gradually going to a maximum dose. There is no risk whatever in using it on children, even infants. In small children, I usually introduce the medicine under the skin, at the back of the neck. The pain inflicted is of no consequence, and there is much less annoyance than pouring it down their throats. The tolerance of the human system to the preparations of opium administered hypodermically, is very surprising, and I shall briefly allude to it. There are several cases on record, well authenticated, of employing it to such an extent that the body was perfectly covered by the points of puncture. There is a young man in this city, who during the past three years has had the instrument used upon himself about *four thousand* times. He had scarlatina when a child, and the inflammation extending from the tonsils and fauces to the eustachian tube resulted in disease and inflammation of the bones of the ear, causing most violent paroxysms of pain. He could not take any of the preparations of opium by the stomach without the greatest distress, and nothing else seemed to give him any relief. The hypodermic syringe was called into requisition and Squibbs' compound liquor of opium, in twenty drop doses, was first used with immediate and marked relief whenever the attacks came on. He visited and consulted the most eminent medical men in the United States, and permanent relief was pronounced by them impossible. After various medication, it was decided to direct remedies alone to the relief of these paroxysms of pain—not supposing the unfortunate young man could much longer survive. Thus he has gone on, using the hypodermic injections daily, generally five or six times in twenty-four hours, for about three years, occasionally increasing the dose, until now, it only requires about one grain of morphine and 1-30 grain of atropine at each injection. He does not seem any worse now than two years since, except some loss of flesh, and a weakening of his intellectual sensibilities, together with an impairment of vision, requiring the use of glasses in reading, produced by the atropine. He carries about his person constantly, wherever he goes, a hypodermic syringe and solution of morphine and atropine, to be used whenever an attack comes on, and so common is it in the city, that any one who first gets to him uses the instrument—even a negro servant who often accompanies him. He carries a little blank book in his pocket, properly ruled, where the time of day and number of drops of the solution are recorded and referred to each time before giving him another injection, as a guide to the amount necessary to be used at that time.

This is one of the most remarkable cases on record of the use of hypodermic injection. How much longer he will survive in this unfortunate condition, no one can tell. He is daily about town, and can walk several miles. It is remarkable that he has not suffered more local trouble from these frequent punctures. They have been made almost exclusively in his arms, from his shoulders to his hands, over and over again, until the areolar tissue is very much thickened and indurated, and has only had an occasional abscess which gave no further trouble after the pus had been discharged.

This case convinces me of the importance and value of the combination of atropine with morphine, when used hypodermically. I find that it does not require such constant and rapid increase of the morphine, and there is less risk of a patient becoming addicted to its habitual use, when thus combined. In view, then, of these facts, and the unlimited calls that may be and are made upon the physician, when the hypodermic use of remedies can be so advantageously employed, is it not highly important that he should always have a standard solution at hand, the strength and effects of which he is well acquainted with, that he may quickly and safeln apply it in all cases of emergency ? I would suggest that every physician have a hypodermic case, which he can either carry in his pocket or medicine chest, containing a syringe with two or three needles and four vials (drachm) filled with the solution of morphine and atropine, Squibbs' compound liquor of opium, Norwood's veratrum viride, and oxalate of cerium—the last to relieve neusea when produced by any of the other medicines, given in doses of four or five grains, on the tongue. I find nothing better. I would not feel that I had performed my duty if, in this connection, I failed to caution my professional brethren concerning the indiscriminate use even of these drugs. Atropia is a powerful poison, the physiological action of which has of late years been the subject of careful experiment and research; the most recent and able writers being Dr. John Harley and Dr. Mennot. In poisonous doses, the frequency of the respiratory movements are *decreased*, through paralysis of the pneumogastric nerves, producing restlessness, insomnia and delirium, until the patient passes into *coma* and dies. The best antidotes are morphia and extract or tincture of calabar bean—and I repeat that the physician who does not use the former, *fails to do his duty*, notwithstanding the conflict of opinion on this subject among our learned professors. I will directly speak more particularly of the calabar bean. Morphia, though rarely deceiving our most sanguine expectations, is not altogether harmless, and may produce frightful symptoms. I will mention a case:

Mrs. H. C., aged 35, in good health otherwise, had been kept awake for seventy-two hours by intense neuralgic pain on the left side of the head, face and neck, arising from a carious molar tooth on left side of lower jaw. She was injected at one A. M., June 28, with one third of a grain of acetate of morphine, dissolved in about four drops of water, under the skin of the left arm just over the insertion of the deltoid muscle. No blood appeared at the puncture. In about fifteen minutes, tightness of the chest and difficulty in breathing were complained of and the patient asked to be raised, saying she felt as if she were dying. Her face and lips now became pale; speech became indistinct (not inaudible); pulse irregular; some spasms of the facial muscles took place, and she fell back, to all appearance, dead. Cold water was freely dashed over her face and chest, and as she was unable to swallow, her tongue was rubbed over with sal volatile, and ammonia applied to her nose—artificial respiration being kept up all the time. During the while, her face was bleached, pulse not to be felt, and respiration not to be perceived. Insensibility continued for about three minutes, then, happily, one or two feeble beats of the pulse, and a shallow inspiration or two, showed returning animation. She then became conscious, pulse feeble and irregular; respiration slow; fingers remained numbed, and both thumbs were firmly drawn into the palms of the hands. This passed off in about six minutes, leaving her feeling very ill, but free from the neuralgic pain, which did not return. There was no feeling of nausea and no attempt at vomiting during any part of the time.

(*To be continued.*)

